# Bone Metastases Are Measurable: The Role of Whole-Body MRI and Positron Emission Tomography

**DOI:** 10.3389/fonc.2021.772530

**Published:** 2021-11-19

**Authors:** Daniela E. Oprea-Lager, Matthijs C.F. Cysouw, Ronald Boellaard, Christophe M. Deroose, Lioe-Fee de Geus-Oei, Egesta Lopci, Luc Bidaut, Ken Herrmann, Laure S. Fournier, Tobias Bäuerle, Nandita M. deSouza, Frederic E. Lecouvet

**Affiliations:** ^1^ Imaging Group, European Organisation of Research and Treatment in Cancer (EORTC), Brussels, Belgium; ^2^ Department of Radiology and Nuclear Medicine, Cancer Center Amsterdam, Amsterdam University Medical Center, Vrije Universiteit Amsterdam, Amsterdam, Netherlands; ^3^ Nuclear Medicine, University Hospitals Leuven, Leuven, Belgium; ^4^ Nuclear Medicine & Molecular Imaging, Department of Imaging and Pathology, KU Leuven, Leuven, Belgium; ^5^ Department of Radiology, Leiden University Medical Center, Leiden, Netherlands; ^6^ Biomedical Photonic Imaging Group, University of Twente, Enschede, Netherlands; ^7^ Nuclear Medicine Unit, IRCCS – Humanitas Research Hospital, Milan, Italy; ^8^ College of Science, University of Lincoln, Lincoln, United Kingdom; ^9^ Department of Nuclear Medicine, University of Duisburg-Essen, and German Cancer Consortium (DKTK)-University Hospital Essen, Essen, Germany; ^10^ Paris Cardiovascular Research Center (PARCC), Institut National de la Santé et de la Recherche Médicale (INSERM), Radiology Department, Assistance Publique-Hôpitaux de Paris (AP-HP), Hopital europeen Georges Pompidou, Université de Paris, Paris, France; ^11^ European Imaging Biomarkers Alliance (EIBALL), European Society of Radiology, Vienna, Austria; ^12^ Institute of Radiology, University Hospital Erlangen, Friedrich-Alexander University Erlangen-Nürnberg, Erlangen, Germany; ^13^ Division of Radiotherapy and Imaging, The Institute of Cancer Research and Royal Marsden NHS Foundation Trust, London, United Kingdom; ^14^ Department of Radiology, Institut de Recherche Expérimentale et Clinique (IREC), Cliniques Universitaires Saint Luc, Université Catholique de Louvain (UCLouvain), Brussels, Belgium

**Keywords:** bone metastases, MRI, PET, measurable, response

## Abstract

Metastatic tumor deposits in bone marrow elicit differential bone responses that vary with the type of malignancy. This results in either sclerotic, lytic, or mixed bone lesions, which can change in morphology due to treatment effects and/or secondary bone remodeling. Hence, morphological imaging is regarded unsuitable for response assessment of bone metastases and in the current Response Evaluation Criteria In Solid Tumors 1.1 (RECIST1.1) guideline bone metastases are deemed unmeasurable. Nevertheless, the advent of functional and molecular imaging modalities such as whole-body magnetic resonance imaging (WB-MRI) and positron emission tomography (PET) has improved the ability for follow-up of bone metastases, regardless of their morphology. Both these modalities not only have improved sensitivity for visual detection of bone lesions, but also allow for objective measurements of bone lesion characteristics. WB-MRI provides a global assessment of skeletal metastases and for a one-step “all-organ” approach of metastatic disease. Novel MRI techniques include diffusion-weighted imaging (DWI) targeting highly cellular lesions, dynamic contrast-enhanced MRI (DCE-MRI) for quantitative assessment of bone lesion vascularization, and multiparametric MRI (mpMRI) combining anatomical and functional sequences. Recommendations for a homogenization of MRI image acquisitions and generalizable response criteria have been developed. For PET, many metabolic and molecular radiotracers are available, some targeting tumor characteristics not confined to cancer type (e.g. ^18^F-FDG) while other targeted radiotracers target specific molecular characteristics, such as prostate specific membrane antigen (PSMA) ligands for prostate cancer. Supporting data on quantitative PET analysis regarding repeatability, reproducibility, and harmonization of PET/CT system performance is available. Bone metastases detected on PET and MRI can be quantitatively assessed using validated methodologies, both on a whole-body and individual lesion basis. Both have the advantage of covering not only bone lesions but visceral and nodal lesions as well. Hybrid imaging, combining PET with MRI, may provide complementary parameters on the morphologic, functional, metabolic and molecular level of bone metastases in one examination. For clinical implementation of measuring bone metastases in response assessment using WB-MRI and PET, current RECIST1.1 guidelines need to be adapted. This review summarizes available data and insights into imaging of bone metastases using MRI and PET.

## Introduction

Bone is a common site of secondary tumor deposits because, in addition to its rigid, calcified, outer cortex, it has a richly vascular inner marrow of bony trabeculae, stroma, haematopoeitic tissue and fat (1). Within bone, it is the crucial balance between osteoblastic and osteoclastic elements that maintains its functional strength and rigidity. Metastatic deposits elicit differential responses from the osteblastic and osteoclastic components, which vary with the type of malignancy and result in strikingly different appearances on imaging (2). In some cases, the tumor incites a predominantly osteoblastic response with a resulting increase in calcified sclerotic matrix, as in prostate and breast cancer (3). In other tumor types, the metastasis causes bony destruction (osteoclastic response) without exciting an osteoblastic response, so that metastases (e.g. kidneys, thyroid, lungs) appear lytic and expansile (3). Finally, the tumor cells can simply invade the marrow without influence on the mineral content of the bone (i.e. radio-occult metastases). In many instances there is a mixture of sclerotic, lytic and radio-occult types. As treatment response is often accompanied by an increase in bony sclerosis (“flare response”), it can be difficult to differentiate it from an osteoblastic response to the tumor itself (4). Moreover, once deformed by the presence of metastases, the rigid form of the bony skeleton does not usually remodel sufficiently after treatment to distinguish untreated from treated tumor. Therefore, on morphological imaging, especially X-ray based, evaluation of response to treatment of bone metastases remains difficult.

RECIST were presented more than 2 decades ago and rely principally on unidimensional size measurements (5). Nowadays, RECIST forms the mainstay of response evaluation of solid tumors to treatment and is universally used in clinical trials of solid tumors. Index lesions with well-defined margins, discernable from adjacent parenchyma are required for reproducible measurements, and specific modifications are set out for some tissues (short-axis measurements for lymph nodes, bi-dimensional measurements for brain lesions). However, because of the blastic response of bone to tumor or to treatment, and of the rigid nature of calcified bone where deformity of the cortex persists after treatment, bone lesions were considered unmeasurable by RECIST. Modifications to RECIST (i.e., RECIST 1.1) stated that bone metastases with soft tissue masses > 10 mm could be considered measurable index lesions (6). Nevertheless, as reduction of the soft tissue component renders the lesions unmeasurable by these criteria again, there remains a critical unmet need for a means of quantifying bone lesions and their response to treatment.

The advent of imaging modalities providing information about tissue microstructure or its metabolism has accelerated the identification of skeletal metastases. ^18^F-fluorodeoxyglucose (^18^F-FDG) PET/CT identifies secondary deposits within bone because of their increased glucose turnover. Its whole-body coverage and increasingly widespread availability has made it of primary importance in cancer staging, particularly in patients where the tumor pathology or molecular profile indicates a high metastatic risk (7, 8). Additionally, techniques such as WB-MRI with DWI have a high sensitivity for identifying highly cellular lesions such as tumors and have been incorporated routinely into the staging of some tumor types such as myeloma (9–11). Dynamic contrast-enhanced MRI (DCE-MRI) for quantitatively assessing vascularization within bone marrow in patients with multiple myeloma was found to be of prognostic significance for these patients (12, 13). While these techniques have their own limitations, they are not hampered by what makes bone lesions unmeasurable by RECIST 1.1 (i.e. radio-occult appearance, sclerotic response and persistent bone deformity on healing). The purpose of this manuscript is to review the MRI and PET techniques available for measuring bone metastases, their opportunities and challenges, and their applicability in various tumor types.

## Different Cancers – Different Types of Bone Metastases

At present, the incidence of bone metastases is 65-75% in advanced metastatic breast cancer, 65-75% in prostate cancer, 60% in thyroid cancer, 30-40% in lung cancer, 40% in bladder cancer, 20-25% in renal cell carcinoma and 14-45% in melanoma (14).

Bone metastases can be classified as osteolytic, osteoblastic, radio-occult, or as a mixed type. Osteolytic metastases are characterized by destruction of normal bone and osteoblastic/sclerotic metastases are characterized by deposition of new bone. Radio-occult lesions have no impact on the mineral content of the bone. Osteolytic lesions are predominantly present in multiple myeloma, renal cell carcinoma, melanoma, non-small cell lung cancer (NSCLC), non-Hodgkin lymphoma (NHL), thyroid cancer, Langerhans-cell histiocytosis and breast cancer, and osteoblastic lesions are present in prostate cancer, neuroendocrine tumors, small-cell lung cancer (SCLC), Hodgkin lymphoma and medulloblastoma (14). Mixed lesions can be found in gastrointestinal cancers and squamous cancers, and 15-20% of bone metastases of breast cancer can be either osteoblastic or mixed (14). Radio-occult lesions can be present in virtually all tumor types. The mechanisms responsible for the impact of metastatic tumor growth on the mineral content of the skeleton are complex and involve the stimulation of osteoclasts and osteoblasts by tumor cells expressing factors. The resulting imbalance between resorption and production of bone matrix subsequently leads to osteoclastic, osteoblastic, or mixed metastatic disease (2).

In osteolytic lesions, bone destruction is primarily mediated by osteoclasts and, in later stages, ischemia can play a role due to the compression of the vasculature (15). Parathyroid hormone-related peptide (PTHrP) induces osteoblasts to produce a receptor activator of nuclear factor κB ligand, which stimulates osteoclast maturation, and thereby plays a critical role in the development of osteolytic lesions. Increased osteoclast activity leads to bone resorption that exceeds the reparative ability of osteoblasts (16). It releases factors from the bone matrix that stimulate PTHrP, thereby creating a vicious cycle. In osteoblastic lesions, osteoblast generation is influenced by transforming growth factor, bone morphogenic proteins (BMP), and endothelin-1 (17). Tumor-derived growth factors stimulate primarily osteoblasts rather than osteoclasts, resulting in deposition of excess abnormal bone. PTHrP can be cleaved by prostate-specific antigen (PSA), resulting in an osteoblastic reaction and decreased bone reabsorption. Furthermore, osteoblast differentiation is influenced by core binding factor alphal, also known as Runx-2 (14). Osteoblast activity may also increase as a reparative process in successfully treated bone metastases, which can be visible on molecular imaging as the so-called “flare phenomenon” and can cause lesions to become denser on radiographs or CT scans (18).

After the tumor cells have left the primary tumor and are in circulation, the bone tumor microenvironment needs to provide a fertile ground (the soil), for the survival and growth of metastatic cancer cells (the seed) (19). Vascular adhesion and extravasation need to occur, and the tumor cells have to remain at the metastatic site. Subsequently, chemo-attractive and adhesion molecules play an important role in the retention of the tumor cells in the bone marrow vasculature. In turn, tumor cells use equivalent molecules, such as chemokines, integrins, osteopontin, bone sialoprotein and type I collagen for organ colonization (20). The microenvironment supports cancer cell survival and growth by producing promoting factors that may contribute to bone metastases development. Subsequently, epithelial-mesenchymal transition occurs, which enables epithelial cells to migrate to a new environment. While this occurs mainly during embryogenesis, in cancer cells this process denotes the invasive phenotype (21).

Sex-associated differences exist in bone metastasis formation from breast-, lung- and prostate cancer. In breast cancer, estrogen influences the bone microenvironment by creating and conditioning a favorable niche for colonization of breast cancer cells. Patients with estrogen receptor α positive (ER+) tumors have bone metastases three times more often than do patients with ER- tumors (22). In lung cancer, it is reported that females more often have bone metastases due to a more favorable bone microenvironment for metastasis formation. In prostate cancer patients, a decrease in the androgen-to-estrogen balance results in bone metastasis formation, with a potentially important role for ERβ that may be similar to that in breast cancer. Androgens as well as estrogens have an influence on osteoblast proliferation and on bone resorbing osteoclasts. In both males and females, estrogens have a dominant effect on bone maintenance and can directly inhibit osteoclasts. Furthermore, androgens directly contribute to male periosteal bone expansion, mineralization, and trabecular bone maintenance (23).

The time from primary diagnosis to the development of bone metastasis can range from months to decades. This implies that tumor cells can lay dormant for significant periods of time after they leave the primary site. It has been shown that the bone is an important reservoir for dormant tumor cells. The best-illustrated cases for clinical dormancy are in breast cancer, where ER+ patients show late recurrences, sometimes decades after removal of the primary tumor. Latent bone metastasis formation likely depends on estrogen regulation, and it is significantly higher in ER+ cases (24).

Bone metastases have unique disease-specific characteristics, such as longevity, fracture healing rates, local and systemic disease progression, and sensitivity to adjuvant treatments. Bone metastases from lung cancer and renal cancer can also show acral distribution (25). Patients with bone metastases of lung cancer historically showed a median survival of approximately 6 months (14). Treatment options for patients with identifiable mutations include immunotherapy and epidermal growth factor receptor tyrosine kinase inhibitors, with evidently improved survival benefit (26). Bone metastases of lung cancer are, in general, sensitive to radiation therapy (27).

The median survival of breast cancer patients with bone-only metastasis is 36 months (28). The medical treatment of breast cancer depends on the hormone receptor and HER-2/neu status and is different for premenopausal and postmenopausal women (25). Furthermore, pain reduction can be achieved, and skeletal-related events and the development of new skeletal lesions can be prevented by the use of bisphosphonates or denosumab, due to their ability to limit bone resorption. Bone metastases of breast cancer are in general radiosensitive, resulting in a lower proportion of surgical treatments (29).

Men with prostate cancer, a good performance status, and bone-only disease have a median duration of disease control after androgen blockade of 4 years and a median survival of 53 months (14). Bone metastases of prostate cancer have a predilection for the axial skeleton, resulting in an increased risk for spinal cord compression (25). However, due to the osteoblastic nature of the metastases, skeletal-related events are relatively uncommon. Also, bone metastases of prostate cancer tend to be radiosensitive, which allows a higher proportion of nonsurgical treatment. In case of a pathologic fracture, healing rates are higher than for most other metastatic carcinomas (29). Treatment with Radium-223, a calcium-mimetic and alpha-emitter that selectively binds to areas of increased bone turnover, results in significantly prolonged OS in patients who had castration-resistant prostate cancer and bone metastases (30).

## Lessons Learned From Experimental Imaging

Quantitative imaging of bone metastases beyond morphology has been studied in preclinical studies on the functional and molecular level using MRI and PET. In these studies, quantitative biomarkers in skeletal lesions were assessed and validated with the underlying histology. Thereby, DCE-MRI parameters in bone metastatic lesions from breast cancer associated with blood volume and vessel permeability were correlated with vessel maturity, while the apparent diffusion coefficient (ADC) from DWI was associated with tumor cellularity as assessed by cell nuclei staining (31). Treatment monitoring in an animal model of osteolytic breast cancer could be performed reliably using DCE-MRI and ^18^F-FDG PET, while therapy response could be detected through functional and metabolic techniques earlier than through morphological imaging (32, 33). Integration of parameters from DCE-MRI and ^18^F-FDG PET by machine learning algorithms enabled the detection of early pathologic processes in the bone marrow preceding morphologic changes in bone structure (34). Thus, parameters from functional and metabolic MR and PET imaging are powerful tools to quantify pathophysiologic processes during colonization of bone marrow and to determine response to treatment of skeletal metastasis.

On the molecular level, PET is the method of choice to determine molecular structures expressed in bone metastases, such as integrins alpha_v_beta_3/5_ or the chemokine receptor CXCR4 (35, 36). Although a major limitation of MRI is the lack of sensitivity when compared to PET, a strategy of signal amplification using a pair of enzymes and an appropriate reducing substrate was presented recently to non-invasively assess epidermal growth factor receptor (EGFR) expression in MRI (37). Besides MRI and PET, other imaging modalities may also be used to determine molecular information in bone metastases, such as ultrasound with its high spatial resolution and unique contrast characteristics of gas-filled microbubbles for enabling the assessment of intra-vascular targets such as vascular endothelial growth factor receptor-2 (VEGFR-2) expressed in bone metastases (38). Thus, molecular imaging strategies for molecular characterization of skeletal lesions have been developed for PET but also for MRI and ultrasound, which are suitable for clinical translation in the near future.

## Magnetic Resonance Imaging (MRI)

### From Axial Spine-MRI to Whole Body-MRI With Diffusion-Weighted Imaging

Since the early 1990s, bone marrow MRI has been developed to overcome the limitations of bone scintigraphy and CT for the assessment of bone metastatic disease, showing an unparalleled sensitivity to the replacement of the bone marrow by neoplastic cells (39, 40).

Axial skeleton MRI (AS-MRI) examinations was first developed as a tool used for the detection of bone marrow replacement by neoplastic foci and their quantification (40, 41). Coverage of the “axial skeleton”, i.e. the whole spine, bony pelvis and proximal femurs, already probes more than 80% of the red marrow containing areas where metastatic disease is observed, and has limited risk to miss isolated peripheral metastatic disease (39, 42).

Whole body MRI (WB-MRI) was later developed for a global assessment of skeletal metastases and for a one-step “all-organ” approach of metastatic disease. The morphologic T1, fat saturated T2/STIR sequences were first used, and were later complemented with functional DWI sequences (42). The “fluid sensitive-fat saturated” T2-like sequences are now preferably acquired using the Dixon method, that not only provides fat-saturated T2 or STIR equivalent “water only” images, but also “fat only” images providing T1-like information and highly sensitive detection of focal lesions on a background of fatty marrow, questioning the residual need for T1 images (43). This T2 Dixon approach can now be extended to whole body examinations: using T2 Dixon sequences as an alternative to the addition of T1 and STIR drastically decreases the acquisition times of anatomical WB-MRI studies (44). Additionally, the Dixon technique offers the possibility to calculate the marrow fat fraction (FF) and generate fat fraction maps. This quantitative approach is gaining interest along with ADC measurements as a biomarker for response evaluation. Indeed, the fat proportion is expected to increase in focal and diffuse marrow infiltration in response to treatment (45).

### Principles, Advantages and Weaknesses

Classic morphologic MRI sequences detect metastases based on the decrease in normal marrow components, mainly fat cell, and on their replacement by neoplastic cells which may present different biochemical composition properties and variable influence on the adjacent bone structure (46).

DWI sequences detect metastatic foci based on the alteration of the movements of water molecules through tissues. In the bone marrow, early infiltration by neoplastic cells is responsible for a decrease in the free movements of water and ADC (47). DWI sequences provide a functional dimension to MRI examinations, as diffusion parameters mainly probe membrane integrity, cell viability and tissue density, and allow a quantitative approach of these parameters. It also largely contributes to the detection and response evaluation in extraskeletal organs involved by the metastatic disease (11, 48, 49).

The detection of neoplastic tissue using MRI does not rely on activation of osteoblasts/clasts and subsequent sclerosis/lysis developed on bone trabeculae, which causes delay in the diagnosis of bone infiltration by radiographs, CT and bone scintigraphy. Unlike PET, MRI does not rely on the avidity of the tumoral tissue for a given radioactive tracer, which largely varies according the primary cancer and also according to the disease stage in the same cancer (50). This provides a “universal” dimension to MRI for the detection and follow-up of metastatic disease.

A major strength of MRI is the detailed morphologic analysis of bones, which allows distinction of benign versus malignant fractures, assessment of extraosseous spread and (sometimes preclinical) impingement on neurologic structures, and monitoring of these complications after initiation of targeted or systemic treatment (51).

As main weaknesses, some benign bone lesions may mimic neoplastic foci and should be identified based on the correlation of DWI and morphologic sequences and on ADC measurements (52, 53). In late stages of the disease, treated lesions and scar tissue within the bone marrow may complicate the detection and size measurements of active metastases, especially on morphologic sequences. DWI sequences and ADC maps then become cardinal for response assessment (54–56).

Another potential limitation of MRI is a benign increase in marrow cellularity of the red bone marrow during the treatment, in response to various factors among which are marrow stimulating drugs, potentially resulting in a diffuse “pseudoprogression” (57). This can be prevented by avoiding the use of MRI during and shortly after the use of these drugs.

### Measurement of Response

Bone marrow MRI is currently used daily in clinical practice and clinical trials to assess the response to treatment of bone only and bone predominant metastatic disease, using several approaches with different complexity (18, 58). Recommendations for a homogenization of MRI image acquisitions and generalizable response criteria have been developed (55). The harmonization of quantitative DWI acquisitions and ADC calculations has been addressed by the United Kingdom Quantitative WB-DWI Technical Workgroup (59).

### Size and Number

**Figure 1:**
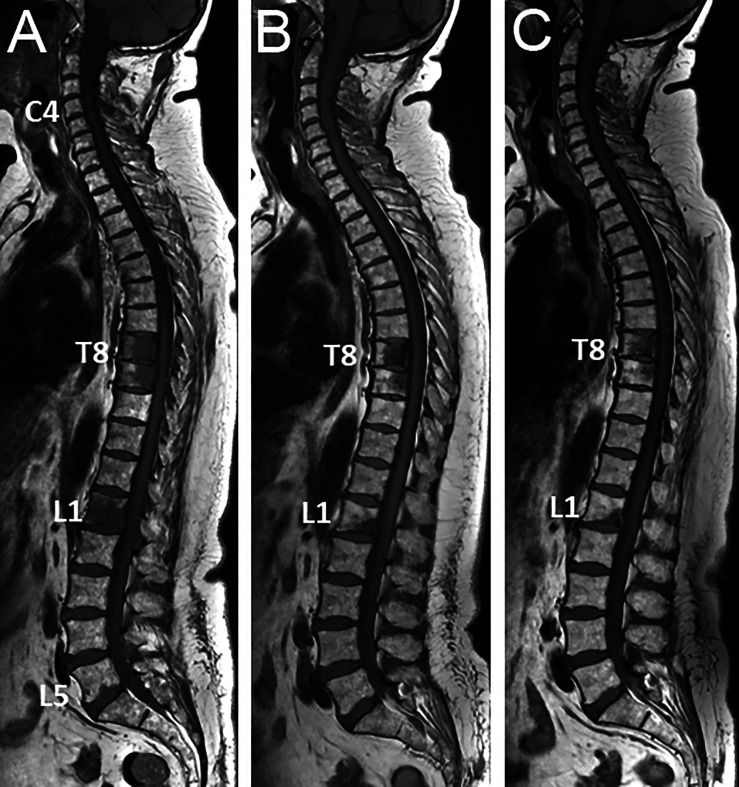
53 year-old woman with newly diagnosed metastatic breast cancer (grade II ductal carcinoma, ER 8, PR8, KI 67 5%, HER2 neu 2+): spinal MRI findings at diagnosis of bone metastases and during treatment. **(A)** Baseline sagittal T1-weighted MR image of the whole spine shows multiple foci of low signal intensity of the bone marrow, typical for bone metastases (posterior arch of C4, vertebral bodies of T8, T9, L1, tiny foci in L5). **(B)** Corresponding MR image obtained 2-m later after combined treatment including a selective estrogen receptor degrader (SERD) and palbociclib shows significant decrease in size of all lesions, and disappearance of the small L5 foci. **(C)** Follow-up MR image obtained 2-m later shows further decrease in size of all lesions, with measurable decrease in lesion size and reappearance of fatty marrow at the periphery and within the lesions, again indicating frank response to treatment.

**Figure 2:**
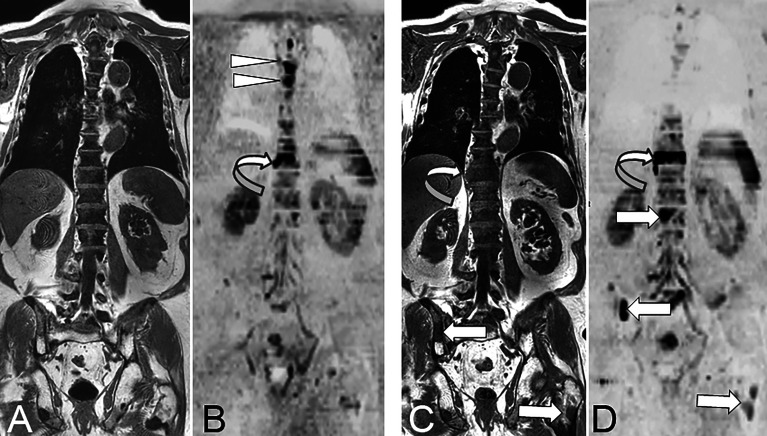
73 year-old man with advanced prostate cancer. Comparison of pre- and post-treatment (enzalutamide) WB-MRI/DWI. Baseline coronal T1-weighted MR image **(A)** shows diffuse bone marrow infiltration within the spine, responsible for diffuse low signal intensity of the bone marrow, and related to advanced metastatic disease after several lines of treatment. The pelvic bones show higher signal of the bone marrow indicating a fatty content due to previous irradiation. Several focal lesions of low signal intensity are visible within the pelvis and left proximal femur. Baseline DWI MR image (**B**; B = 1000 s/mm^2^, inverted grey scale) shows high signal intensity foci typical for active bone metastases within the T4, T5 (arrowheads) and T10 (curved arrow) vertebrae. Follow-up T1-weighted MR image **(C)** shows no evident change of the spinal bone marrow, but increase in the right paraspinal extension of the T10 metastasis (curved arrow), and a new lesion within the right posterior iliac crest (arrow). Follow-up DWI MR image **(D)** shows disappearance of the midthoracic vertebral lesions, but increase in size and right paraspinal extension of the T10 vertebral lesion (curved arrow), and appearance of new lesions within the L1 vertebral body, the right iliac crest and left proximal femur (arrows). The observation of concurrent signs of disease response and progression is frequent, especially in advanced stages of metastatic cancer.

Metastatic disease to the bone marrow may present as a focal or a diffuse pattern. Evolution from a normal appearing marrow to a focal or diffuse pattern, increase in number and size of focal lesions will indicate disease progression (60). A decrease in focal lesion number and size, return from diffuse or focal patterns of marrow infiltration to a normal marrow appearance will indicate response (**Figures 1**, **2**).

RECIST-like criteria can be transposed to bone marrow metastases. Simple size measurements of bone metastases on morphologic sequences in (a limited sample of) bone metastases allows objective assessment of response, especially in early disease. This approach can be used on morphologic sequences and on high b value DWI sequences. In prostate cancer, this approach more than doubles the proportion of patients with measurable metastatic disease, previously limited to those patients with quantifiable abdominal lymph nodes (41).

### Non-Quantitative Features

Additional “qualitative” signs may be used for response assessment on MR images. The progressive appearance of a “fatty halo” of high signal on T1-weighted images at the periphery of regressing focal lesions indicates responsive disease (60). Conversely, the disappearance of a peripheral “cellular” of high signal intensity on T2-weighted images, representing active or aggressive disease, also represents an early sign of response, whereas its re-appearance suggests disease relapse. The appearance of malignant vertebral compression fractures, and appearance/progression of extraosseous/epidural spread unambiguously indicate progressive disease (60).

### Quantitative Functional and Multiparametric Approaches

The quantitative approach can be directed either to individual lesions or to the whole-skeleton using ADC measurements and mapping derived from DWI sequences and fat fraction (FF) measurements derived from Dixon acquisitions. This approach becomes cardinal in advanced metastatic disease where previously treated lesions and scar tissue complicate the size measurements of active lesions on morphologic sequences.

Response to treatment is associated with an early increase in ADC values within individual lesions (61). At a later stage, responsive bone metastases present a decrease in ADC values together with a decreased signal on high b-value images due to recolonization by normal bone marrow. A sharper decrease in signal intensity and ADC is related to the sclerotic transformation of treated lesions, which is also observed on anatomic sequences. A total diffusion volume can be derived from WB DWI sequences for a global quantification of the metastatic burden and its follow-up under treatment (62, 63). The FF presents an early increase in focal and diffuse metastatic infiltration in response to treatment) (45).

Multiparametric MRI by definition combines anatomical and at least two functional sequences. The multiparametric WB-MRI approach used for the quantitative evaluation of bone lesions combines anatomical T1 and STIR sequences (potentially replaced by single T2 Dixon acquistions), FF measurements, and functional DWI sequences along with ADC maps.

The METastasis Reporting and Data System for prostate cancer (MET-RADS-P) guidelines were designed in prostate cancer, in an international initiative to standardize WB-MRI protocols and most importantly to provide multiparametric response evaluation criteria for bone, node, and visceral lesions (55). These criteria combine quantitative approaches of ADC and FF within bone marrow metastases, RECIST-like size criteria transposed to bone lesions, and RECIST criteria for node and visceral lesions follow-up. They allow categorization of the disease response or progression on a 5-point Likert scale. The method also offers the possibility to record the heterogeneity of response within metastases and categorizes the response as “discordant” if some bone lesions or soft-tissue are progressing, while others are stable or are responding, and vice-versa. The reproducibility of the technique as well as its use by readers with various experience have been validated (64). The same criteria may be transposed for WB-MRI studies performed for lesion follow-up and response assessment in bone-only or bone-predominant metastatic disease from other primary cancers.

### Target Cancers

The objective parameters extracted from AS-MRI and WB-MRI/DWI are increasingly used to assess response of bone metastases to treatment in a large number of primary cancers.

In prostate cancer, AS-MRI and later WB-MRI were introduced after demonstration of their superiority to bone scintigraphy for detection of bone metastases and for a one step staging of bone and lymph node involvement (40, 65, 66). The current roles of WB-MRI to assess metastatic disease have been recently illustrated and compared to other techniques (44). PSMA-PET/CT is most likely the current most sensitive technique for the detection of low volume metastatic disease and for therapeutic decision (curative versus systemic treatment) in newly diagnosed prostate cancer and at the biochemical recurrence stage. WB-MRI is an optimal non-irradiating alternative for polymetastatic disease detection and follow-up under systemic treatment (**Figure 2**). WB-MRI might become the first choice in advanced disease, castration-resistant prostate cancer (CRPC), as PSMA-PET/CT might be confounded by androgen blockade (AB) treatments which induce short term upregulation of PSMA expression and long term downregulation of this expression, limiting the possibility of following metastatic prostate cancer lesions at this stage (67, 68).

In breast cancer, AS-MRI and WB-MRI were also introduced to overcome the limitations of bone scintigraphy (BS) and CT for the detection of bone metastases and evaluation of their response to treatment (**Figure 1A**) (54, 69, 70). WB-MRI progressively becomes a key imaging modality for the evaluation of response in bone only/predominant metastatic breast cancer for the follow-up of treatment response (71). In patients with advanced breast cancer treated with systemic treatment of metastatic disease and followed-up with WB-MRI in addition to other imaging modalities (CT, BS, TAP-CT or PET/CT), WB-MRI discloses progressive disease earlier than the reference examination and provides decisive information for changes in treatment in more than 50% of patients (72–74). Of note, WB-MRI shows a frequent discrepancy between response as assessed locally within the primary cancer and within metastases, and disease progression is identified earlier in distant disease compared to local disease assessment (75).

There is a consistently increasing number of indications of WB-MRI for bone and visceral metastases detection in various primary cancers, sometimes relying on the design of disease- or patient- tailored MRI studies (coverage of lung, liver, and brain, with specific sequences according to primary cancer). WB-MRI can for example be proposed in this indication in lung, thyroid, kidney and colorectal cancers, in melanoma, myxoid liposarcoma, Ewing sarcoma or osteosarcoma. The detection of bone metastases using the same technique substantiates its use for the subsequent evaluation of the response of bone lesions to treatment (76).

## Positron Emission Tomography (PET)

### Quantitative Assessment of Bone Metastases on PET

Traditionally, PET is used for staging of many cancer types because of its high sensitivity for visual detection of metastatic disease, typically using ^18^F-FDG as radiotracer. In 2009, novel qualitative and quantitative approaches to metabolic tumor response assessment, solely applicable for ^18^F-FDG PET, were proposed (77). The purpose was to overcome the limitations of morphologic imaging alone-based criteria (e.g. RECIST, RECIST1.1) and to capitalize the benefit of using newer cancer therapies. The framework for PET Response Criteria in Solid Tumors (PERCIST), version 1.0, was meant to serve as an example for use in clinical trials and in structured quantitative clinical reporting (77).

In current practice, however, the quantitative nature of PET is often unexploited. Especially in the case of bone metastases that are deemed non-measurable by RECIST 1.1, quantification of radiotracer uptake might prove crucial for assessing bone disease through changes in the viability or molecular processes of tumor cells instead of lesion morphology. A further advantage is that quantitative PET assessment can be performed on a per-lesion basis, as well as on a whole-body level.

Parameters that can be extracted from routinely acquired static whole-body PET images have been validated for many tracers in different cancer types (78–82). In general, these parameters can be divided into those based on (83): i) tracer uptake intensity (e.g. standardized uptake values, SUVs), ii) metabolically active tumor volumes (MATV), and iii) a combination of both, representing the total tracer uptake in a tumor. Typical SUV metrics are the mean uptake (SUV_mean_), the maximum uptake (highest voxel value; SUV_max_), or the peak uptake (highest average value of a 1cm³ sphere; SUV_peak_) within an identified lesion. Depending on specific radiotracer kinetics, uptake may need to be normalized to background activity in e.g. liver or blood (81). Metrics combining lesion volume and tracer uptake, such as total lesion glycolysis (TLG) for ^18^F-FDG, seem especially promising for objective longitudinal assessment of bone metastases load, as they provide information on the total amount of viable tumor tissue within a bone lesion both on an individual lesion and patient-basis (84, 85).

### Target Cancers

#### Prostate Cancer

**Figure 3:**
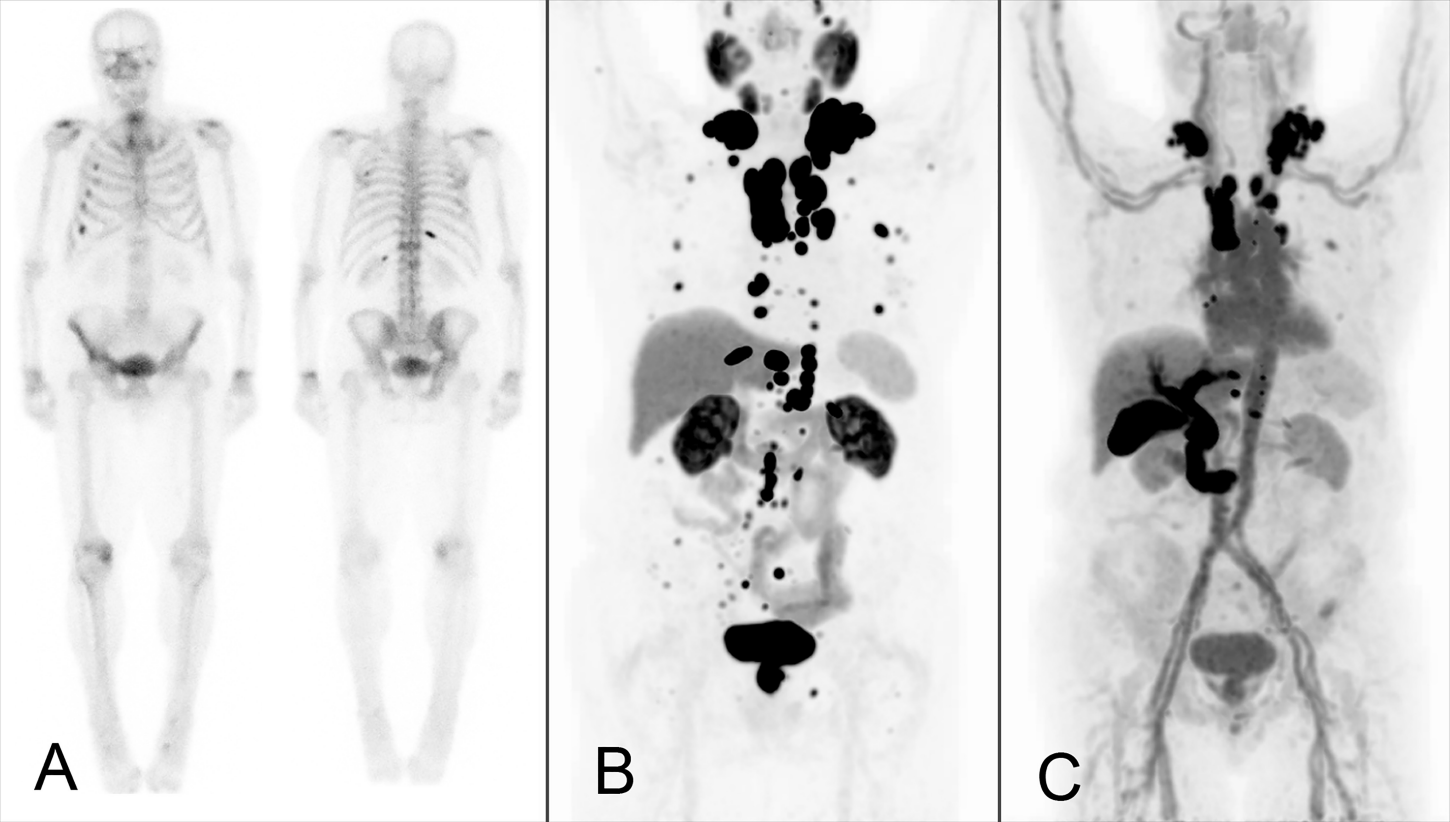
Example of a male patient with bone and lymph node metastases from castration-resistant prostate cancer who underwent bone scintigraphy **(A)** and ^18^F-DCFPyL **(B)** and ^18^F-FDHT PET **(C)** for research purposes. Bone scintigraphy demonstrated several rib metastases. A large number of additional (measurable) bone metastases were observed on ^18^F-DCFPyl PET, with additional lymph node metastases detected on-par. Disconcordant AR-expression visualized on ^18^F-FDHT PET.

In metastatic prostate cancer, osteoblastic or mixed bone lesions with minor soft tissue component are frequently observed, challenging accurate RECIST1.1-based follow-up for these patients. With the recent introduction of several PET-tracers targeting the PSMA (**Figure 3**), detection of prostate cancer lesions has significantly improved (86, 87). In 2018, guidelines for standardized interpretation of PSMA PET images (PROMISE) were proposed (88). Quantitative parameters for evaluation of treatment response using PSMA PET/CT, besides well-known maximum standardized uptake values (SUV_max_), have been proposed including PSMA tumor volume (PSMA-TV) and total lesion PSMA expression (TL-PSMA) (85). Initial studies evaluating metrics such as PSMA-TV and TL-PSMA for metabolic response assessment have shown promising results, some of these through a ‘PSMA-modified’ RECIST or PERCIST classification system. Importantly, several studies reported an association of these PSMA PET parameters with overall survival (OS) during treatment with radioligand therapy (RLT) with ^177^Lu-PSMA (89–91). A recent systematic review summarized the available evidence for using quantitative PSMA parameters versus serum PSA in assessing response for castration-resistant prostate cancer (92).

In parallel to ER-targeted PET imaging in breast cancer with 16α-^18^F-fluoro-17β-estradiol ([^18^F]FES), androgen receptor (AR)-targeted PET imaging in prostate cancer is possible using ^18^F-fluorodihydrotestosterone (^18^F-FDHT; **Figure 3**), which binds the intracellular AR in prostate cancer cells (93). This enables quantitative assessment of AR-expression in bone metastases, both for response monitoring and prognostic purposes (80, 93, 94). ^18^F-FDHT cannot be used during treatment with drugs that directly block the AR (95, 96). For PSMA-ligands and ^18^F-FDHT, technical validation studies assessing tracer pharmacokinetics and repeatability have been performed, enabling their clinical use in (quantitative) response assessment (80, 81, 94, 97, 98).

The PET response assessment approach for bone metastases in prostate cancer using PSMA PET may be extended to other targeted PET tracers, such as ^18^F-NaF and ^18^F-FDHT in prostate cancer, ^18^F-FES in breast cancer and ^18^F-FDG and ^68^Ga-fibroblast activation protein inhibitors (^68^Ga-FAPI) in a multitude of cancer types (94, 99–102).

#### Lung Cancer

The skeleton is the most common site of distant metastasis in lung cancer. Approximately 30% to 40% of the patients with advanced cancer will develop bone metastases, which represent 10% of disease recurrence even in early stage operable lung cancer (15, 103, 104). ^18^F-FDG PET/CT plays a key role in the diagnostic work-up of lung cancer, being fundamental especially at diagnosis and during staging/restaging (105). Consequently, all clinical guidelines support the use of the modality for the assessment of advanced disease (106–110), given the high diagnostic accuracy in depicting distant metastases for which ^18^F-FDG PET/CT results superior to other conventional imaging (111–115).

Recent meta-analysis data comparing [^18^F]FDG PET/CT with WB-MRI show similar performances for staging NSCLC, i.e. area under the curve (AUC) 0.95 for PET versus 0.93 for MRI (116). The performance was also similar in case of SCLC patients (117). When considering only bone metastases, dedicated meta-analyses in lung cancer have proven PET/CT is superior to other modalities, with a pooled sensitivity for [^18^F]FDG PET/CT, MRI and bone scintigraphy (BS) of 92%, 77% and 86%, respectively, associated to a pooled specificity of 98%, 92% and 88%, respectively (118). Depending on cancer type, there is also an associated impact in patient management that ranges from 12%-40% of the cases (105, 111, 115, 118).

#### Breast Cancer

The use of ^18^F-FDG PET/CT in breast cancer faces more conflicting indications based on major clinical guidelines (111, 119, 120). While staging in advanced or suspicious metastatic breast cancer is widely supported, initial preoperative staging is regarded of limited value. Still, the results of a recent meta-analysis in 4276 patients prove that the use of ^18^F-FDG PET for initial evaluation of breast cancer leads to a change in staging and management in 25% and 18% of patients, respectively (121). With younger age, clinical stage III to IV and histologic grade II to III were significantly associated with a greater proportion of changes. These results are most likely attributable to the superior diagnostic accuracy of ^18^F-FDG PET/CT compared with other modalities (122, 123). In particular, the pooled sensitivity and specificity of whole-body ^18^F-FDG PET and PET/CT are reported to be 99% and 95%, respectively, compared to 57% and 88% for conventional imaging studies (8, 124).

Approximately 70%–80% of breast cancers express hormone receptors (HR), i.e. ERα and/or progesterone receptors (PR) (125). Thanks to the use of [^18^F]FES PET, breast cancer metastases can be characterized non-invasively also for ER status reaching a pooled sensitivity and specificity of 78% and 98%, respectively (126, 127). The information obtained by [^18^F]FES PET can be used also to predict the response to hormonal therapy in patients with locally advanced or metastatic breast cancer. For this purpose, SUV cut-off values can be applied, for example 1.5 and 2.0, demonstrating pooled sensitivities and specificities for response prediction of 63.9% vs. 66.7%, and 28.6% vs. 62.1%, respectively (127). In newly diagnosed ER-positive breast cancer, moreover, [^18^F]FES PET shows a sensitivity of 90.8% versus 82.8% for ^18^F-FDG PET/CT, thus potentially leading to a change in patient management in 26.3% of the cases (128).

Besides overexpression of hormone receptors, a proportion of breast cancer tumors is known to show expression of human epidermal growth factor receptors 2 (HER2) (129). In recent years, whole body HER2-targeted PET imaging has proven to be a valuable tool, both for the identification of patients suitable for anti-HER2 therapy and monitoring therapeutic efficacy (130–135). HERs can be targeted by several inhibitors that directly block the receptors on HER-expressing tumor cells or interfere with their signaling pathways (135). HER2-targeted PET imaging with ^64^Cu- or ^89^Zr-labeled antibodies is effective but typically requires late time points acquisitions due to the antibody and radio-isotope properties (132, 133). ^68^Ga-labeled affibody molecules targeting HER2 allow for routine same-day PET imaging, thereby improving the clinical utility of HER2-targeted imaging, and have yielded promising initial results (130, 131, 136). More clinical data on the use of HER2-targeted molecular imaging in breast cancer patients is required before future clinical use.

### Challenges and Opportunities in PET

Absolute measurements of tumor lesion PET metrics are inherently dependent on the method used for tumor delineation (137). Several segmentation methods have been proposed, most semi-automatic and relatively easy to apply, requiring a good repeatability and reproducibility basis in order to detect small changes during response monitoring (138, 139). Software packages are often vendor-supplied and differences between several methods have been well evaluated (139–141).

Evaluating longitudinal changes in tracer uptake on PET typically requires patients to be scanned on preferably the same PET/CT system using the same image reconstruction protocol (142–144). Still, in PET the technical uncertainties can be easily mitigated by harmonization of PET/CT system performances between and within clinical centers. The latter is achieved by the EARL accreditation program showing that harmonization is feasible and is a prerequisite for a high reproducibility of quantitative reads (145, 146).

Besides technical challenges, biological aspects need to be considered when using PET for measuring bone lesions and response to treatment in a clinical setting. The optimal timing of disease assessment will depend on the specific treatment type a patient is receiving, such as radiotherapy, RLT, chemotherapy, or other targeted drugs. For example, systemic cytotoxic or antihormonal treatments may elicit so called ‘flare’ phenomena, potentially precluding the use of PET early during treatment follow-up (147–150). This can be avoided by adhering to clinical guidelines and not performing PET too soon after treatment initiation.

Recent and ongoing technical advances have given rise to several new opportunities in PET imaging. PET initially was a stand-alone modality, but has moved on to become a hybrid imaging modality (with CT and MRI). Even more recently, the novel ‘total body’ PET systems have become available (151, 152). These total body (or ‘long axial field of view’) PET systems can be used to perform PET imaging in a single field-of-view instead of multiple bed positions, with typical FOV from skull apex to mid-thighs (151–153). Not only does this severely shorten the required acquisition time (a large benefit for patients with often painful bone metastases), but this is also accompanied by a large increase in system sensitivity which is expected to improve lesion detection rates (153). Moreover, the total body PET might enable quantitative parameters incorporating radiotracer dynamics, such as whole body Patlak (154), to be extracted and parametric images to be generated.

Advances in computer science have made the routine use of artificial intelligence (AI) in medical imaging analysis possible (155). A common application of AI in PET lies in the analysis and modeling of radiomics features. Radiomics pertain to large volumes of data on tumor shape, size, metabolism and texture that can be extracted from PET-positive lesions, providing an image-based tumor phenotype (155–157). The Imaging Biomarker Standardization Initiative has harmonized performance of radiomics software packages to allow for its robust and reproducible use (157). Recently, consensus recommendations for considerations on the use of radiomics (both PET, CT, and MRI) in clinical trials have been proposed (158). Deep learning techniques, which do not require extraction of predefined features seem particularly promising for segmentation purposes of PET-avid bone metastases (159, 160). For PSMA PET, a deep learning algorithm for automated analysis of PET images (‘aPROMISE’) has been developed (161).

## Hybrid Imaging (PET/MRI)

The unique potential of hybrid imaging, as reviewed by Schmidkonz and colleagues, lies in the assessment of complementary parameters on the morphologic, functional, metabolic and molecular levels of bone metastases from different modalities in a single examination (162). When combining PET with CT in a PET/CT study, the CT component enables assessment of bone morphology and osseous destruction, while MRI in a PET/MRI hybrid study will offer superior soft tissue contrast. Due to the (still) novelty and increased cost and complexity of PET/MRI, this technique currently is primarily compared to PET/CT for assessing the respective potential of these two imaging approaches for evaluating bone metastases.

When comparing the performance of ^18^F-FDG PET/CT with ^18^F-FDG PET/MRI for the assessment of malignant bone lesions, the overall performance of PET/MRI has been found to be equivalent to PET/CT for the detection and characterization of bone lesions when these hybrid techniques were performed sequentially (163). However, in PET/MRI, lesion delineation and allocation of PET-positive findings were found to be superior to PET/CT (163). Samarin and colleagues reported similar results from a comparison of ^18^F-FDG PET/CT with ^18^F-FDG PET/MRI in 24 patients with bone metastases from different primary tumors (164). The overall detection rate was not significantly different between PET/CT and PET/MRI, but the latter provided higher reader confidence and improved conspicuity as compared with PET/CT (164).

In a prospective comparison of the diagnostic accuracy of ^18^F-FDG PET/MRI and CT, PET/MRI was significantly better than CT for the detection of bone metastases in patients with newly diagnosed breast cancer (165). Also, in a particular series of 109 breast cancer patients, PET/MR demonstrated an improved sensitivity over ^18^F-FDG PET/CT alone, where the sensitivity of PET/MR and PET/CT were 96% and 85%, respectively (166). In men with biochemical recurrence of prostate cancer following curative therapy, ^68^Ga-PSMA-11 PET/MRI demonstrated a high detection rate especially for recurrent disease with low PSA values, but included all sites of local or distant recurrence including lymph nodes and bone (167). In 26 patients with prostate cancer, ^68^Ga-PSMA-11 PET/MRI and PET/CT performed equally regarding the PET component for detection of bone metastases, while two PET-positive skeletal metastases could be confirmed on contrast MRI, but not on CT (168).

An interesting approach for patients with both osteolytic and osteoblastic metastases from breast or prostate cancer was proposed by Sonni and colleagues (169). Combining ^18^F-FDG and Na^18^F in PET/MRI was superior for the detection of skeletal metastases as compared to whole-body bone scintigraphy (169). This approach includes in an innovative manner both a radiotracer (Na^18^F) for the assessment of primarily osteoblastic activity in osteoblastic lesions, and another tracer (^18^F-FDG) for assessing increased glucose metabolism in the soft tissue component of predominantly osteolytic metastases. Based on the data and results referenced above, PET/MRI appears rather superior to PET/CT for the detection of metastatic bone lesions, but it lacks the morphologic information of bone and osteoblastic bone formation derived from the CT component, which might be mitigated some through innovative approaches as reported by Sonni and co-workers.

## Beyond RECIST and PERCIST

In 2009, the PET Response Criteria in Solid Tumors (PERCIST) were introduced for ^18^F-FDG PET (77). Later on, along with the detailed describing of the ^18^F-FDG PET requirements to allow quantitative expression of the changes in PET measurements and assessment of overall treatment response, a Simplified Guide to PERCIST 1.0 was published (170). The PERCIST criteria enable avid bone target lesions to be selected based on their metabolic activity, and response to be measured objectively based on the changes in metabolic activity even in the absence of an evident anatomic change. PERCIST, however, only considers the change in uptake of a single target lesion when assessing response, which is the lesion with the highest SUV_peak_ value normalized for lean body mass (SUL_peak_). New lesions, in the bone or elsewhere, result in progressive disease by definition. The target lesion may or may not be within the bone, but all bone lesions have to be considered in the selection of target lesion. Of note, there is no impact of changes in volume of lesions, only the uptake concentration is considered. Compared to RECIST, PERCIST represents a major step forward for bone assessment as it considers bone lesions equally to any other lesions anywhere else in the body.

The PERCIST approach focusses on the remaining hottest lesion and has similarities with the therapy response criteria for lymphoma, where the most active remaining lesions play a dominant role (171). This “hottest lesion” centric approach is well tailored to therapies with curative intent, but it might miss a beneficial effect in non-curative therapies, where tumor control and tumor bulk reduction are clinically relevant achievements. A recent approach in image analysis is about abandoning the selection of target lesions, as determined on baseline or post-therapy scans, and aiming to take the entire tumor bulk in consideration. The high contrast of modern oncological PET tracers [e.g., ^18^F-FDG, somatostatin receptor (SSTR) and PSMA ligands, ^18^F-DOPA, ^18^F-MFBG (172)] permits straightforward three-dimensional segmentation of lesions; by segmenting all lesions, the total tumor burden can then be obtained. This type of analysis does not distinguish between bone and non-bone lesions and thus puts bone metastases on par with other metastases.

There is evidence that the baseline metabolic tumor volume (MTV) is an important prognostic factor, e.g. in NHL, NSCLC and multiple myeloma, as well as in prostate cancer patients treated with the bone-seeking agent radium-223 (173–176). Furthermore, MTV can be combined with metrics of tumor distance within a patient to not only represent volume, but also dissemination for better reflecting prognosis, as shown in NHL (175). Evaluation of the changes in MTV and/or TLG have been shown to outperform PERCIST-based approaches in tumors with frequent bone lesions, such as Ewing sarcoma and osteosarcoma (177–180). Volumetric determination on PET is not hampered by bone/soft tissue interfaces, taking the total tumor burden into account in combination with the metabolic activity. Total tumor burden analyses can be combined with specific organ segmentation either based on PET or CT, e.g. for spleen (for lymphoma) and bone, to generate organ-specific tumor burden (181, 182). Furthermore, the segmentation leading to total tumor burden or organ-specific tumor burden can also be used as a mask to determine specific radiomic features, which can provide even more information for response evaluation (180).

Although promising, some challenges remain to the application of tumoral volumes for routine therapy response monitoring: (i) lack of standardization of uptake thresholds for PET-positive tumor delineation; (ii) still too time consuming for clinical routine; (iii) no prospectively defined response criteria. Especially regarding i and ii, it is expected that advances in tumor segmentation, e.g. with further automatization of the segmentation process and contributions from deep learning-based AI algorithms, will increase the robustness, accuracy and feasibility of total tumor segmentation to routine clinical practice levels (141, 183). Based on results from current and ongoing studies, automated tumor segmentation should actually be one of the key expected improvements from AI applications to PET (and other) imaging (160).

At any rate, similar analyses can be developed for non-FDG tracers, e.g. with SSTR ligands in neuroendocrine tumors and PSMA ligands in prostate cancer, Na^18^F in breast and prostate cancer patients (184–187). For evaluation of response on PSMA PET, consensus criteria have recently been proposed with specific cut-off values for both uptake and volume (188).

A specific case of total tumor burden imaging that is worthy to mention is the use of the bone scan index (BSI), which is a metric based on 2D planar bone scintigraphy that reflects the fraction of bone showing increased turnover due to metastatic invasion (189). It has been proposed two decades ago as a metric for tumor burden and response assessment in metastatic prostate cancer (189). Changes in BSI under treatment have been shown to correlate with OS in patients with metastatic castrate-resistant prostate cancer (mCRPC) treated with a range of therapies (190). This has been corroborated in multicenter trials in mCRCP patients treated with abiraterone acetate and with radium-223 dichloride (191, 192). Although promising, it is expected that the shift from 2D to 3D imaging and the increasing use of novel PET tracers that can pick up lesions outside of the bone as well as bone lesions (e.g. PSMA ligands) will eventually displace the currently widespread adoption of BSI for therapy response. Accordingly, similar but PET-based metrics from PSMA and/or Na^18^F PET will likely outperform and replace BSI.

## Conclusion

Modern imaging with PET and MRI allows bone metastases to be detected and assessed both before and after therapy, without the drawbacks of X-ray based imaging techniques (e.g. radiographs, CT). These techniques assess bone metastases within the same framework, as metastases in other organs. They further allow total tumor burden to be assessed within a single imaging session, and also the development of response criteria that include the bone, thus filling a critical gap in the RECIST1.1 framework. The EORTC, PERCIST and recent PSMA PET criteria are examples of criteria that take bone metastases in consideration, on-par with extra-osseous lesions. PET and/or MRI can detect and characterize bone metastases of various types (e.g. lytic, sclerotic, radio-occult or mixed) independently from the bone density changes. In contrast with CT, they are not affected by changes in bone mineralization induced by the tumor(s), and are not dependent on soft-tissue components (as required by RECIST 1.1). Whole body MRI including modern techniques such as DWI, DCE-MRI and mpMRI can provide both detailed information on anatomical structures as well as functional information on individual lesions and whole body tumor burden. Modern PET imaging is performed on hybrid cameras, with CT (from PET/CT) allowing assessment of the bone mineral content (including fractures), while MRI (from PET/MRI) can more often clarify a correlate for the lesions observed on PET. Total tumor burden, incorporating bone metastases on par with other metastases, is an attractive approach to be applied in most PET tracers. While advances in algorithms and deep-learning contributions are expected to permit the determination of total tumor burden metrics in actual clinical routine before and after therapy, response criteria through total tumor burden assessment are currently developed, taking into consideration the tracer, therapy and underlying cancer type.

## Author Contributions

All authors listed have made a substantial, direct, and intellectual contribution to the work, and approved it for publication.

## Conflict of Interest

KH reports personal fees from Bayer, personal fees and other from Sofie Biosciences, personal fees from SIRTEX, non-financial support from ABX, personal fees from Adacap, personal fees from Curium, personal fees from Endocyte, grants and personal fees from BTG, personal fees from IPSEN, personal fees from Siemens Healthineers, personal fees from GE Healthcare, personal fees from Amgen, personal fees from Novartis, personal fees from ymabs, all outside the submitted work. CD reports consultancy for Sirtex, Terumo and PSI CRO, speaker fees from Terumo and Advanced Accelerator Applications and is a member of advisory board for Terumo and Ipsen. EL reports grants from Fondazione AIRC and Italian Ministry of Health, royalties from Springer, lecturer fees from MI&T congressi and ESMIT. LF reports speaker fees from Sanofi, Novartis, Jannssen, and General Electric, congress sponsorship from Guerbet, industrial grant on radiomics from Invectys and Novartis, and co-investigator in grant with Philips, Ariana Pharma, Evolucare.

The remaining authors declare that the research was conducted in the absence of any commercial or financial relationships that could be construed as a potential conflict of interest.

## Publisher’s Note

All claims expressed in this article are solely those of the authors and do not necessarily represent those of their affiliated organizations, or those of the publisher, the editors and the reviewers. Any product that may be evaluated in this article, or claim that may be made by its manufacturer, is not guaranteed or endorsed by the publisher.
